# Organizational readiness for wellness promotion – a survey of 100 African American church leaders in South Los Angeles

**DOI:** 10.1186/s12889-019-6895-x

**Published:** 2019-05-17

**Authors:** Annette E. Maxwell, Rhonda Santifer, L. Cindy Chang, Juana Gatson, Catherine M. Crespi, Aziza Lucas-Wright

**Affiliations:** 1 University of California Los Angeles Fielding School of Public Health & Jonsson Comprehensive Cancer Center; UCLA Kaiser Permanente Center for Health Equity, 650 Charles Young Dr. South, A2-125 CHS, Box 956900, Los Angeles, CA 90095-6900 USA; 2 Division of Cancer Research and Training, Charles R. Drew University of Medicine and Science, Los Angeles, CA USA

**Keywords:** African American churches, Survey of senior pastors, Readiness assessment, Resources and barriers to implement wellness activities

## Abstract

**Background:**

Churches are an important asset and a trusted resource in the African American community. We needed a better understanding of their readiness to engage in health promotion before launching a large-scale health promotion effort in partnership with South Los Angeles churches.

**Methods:**

In 2017, we conducted surveys with leaders of 100 churches. Surveys were conducted face-to-face (32%) or by telephone (68%) with senior pastors (one per church) and lasted on average 48 min. We compared small (less than 50 active members), medium (50–99 active members) and large churches (at least 100 active members), and assessed which church characteristics were associated with the implementation of wellness activities.

**Results:**

Medium and large churches conducted significantly more wellness activities than small churches and were more likely to have wellness champions and health policies. Regardless of church size, insufficient budget was the most commonly cited barrier to implement wellness activities (85%). A substantial proportion of churches was not sure how to implement wellness activities (61%) and lacked volunteers (58%). Forty-five percent of the variation in the number of wellness activities in the last 12 months was explained by church characteristics, such as size of congregation, number of paid staff, leadership engagement, having a wellness ministry and barriers.

**Conclusions:**

Many churches in South Los Angeles are actively engaged in health promotion activities, despite a general lack of resources. We recommend a comprehensive assessment of church characteristics in intervention studies to enable the use of strategies (e.g., stratification by size) that reduce imbalances that could mask or magnify study outcomes. Our data provide empirical support for the inner settings construct of the Consolidated Framework for Implementation Research in the context of health promotion in African American churches.

**Electronic supplementary material:**

The online version of this article (10.1186/s12889-019-6895-x) contains supplementary material, which is available to authorized users.

## Background

California has the largest population of African Americans in the western United States, and a large proportion of African Americans are living in South Los Angeles (27% of the population, compared to the state average of 8%). South Los Angeles, a region containing 28 neighborhoods, has the highest rate of obesity in the Los Angeles area and high mortality due to diabetes, coronary heart disease, stroke and lung, breast, cervical and colorectal cancer. These health disparities exist in a setting of high rates of poverty and limited access to care. More than 33% of the population in South LA have a household income below 100% federal poverty level (18% in LA County), 42% of adults have less than a high school education (22% in LA County), and one third of adults report difficulty accessing medical care (24% in LA County) [1].

In the African American community, which has been marginalized and mistreated in biomedical research, churches are trusted sources of information and support [2]. Many religious leaders are interested in addressing the physical health needs in their community in addition to providing spiritual and social support [3]. Many churches have health ministries that are dedicated to improving the overall health of their members. Church-based health promotion programs are effective, feasible and acceptable in many minority populations including African Americans and in populations that have limited access to health promotion programs [4–9].

Churches often conduct health programs through *community health advisors (CHAs)*, trained lay people who are well known and respected by other church members. CHAs can serve as referral source and role models, can provide counseling and print materials, and can advocate on behalf of community members [8, 10–13]. However, even when churches partner with academic institutions and are provided with an evidence-based health promotion program, CHAs often need support to fully implement program components and to sustain program activities [14–17].

While recognizing the important role that churches can play in encouraging members of the African American community to engage in health promotion, we decided that we needed a better understanding of their readiness to engage in health promotion before launching a large-scale health promotion effort in partnership with South LA churches. Capacity and readiness have sometimes been used interchangeably. Rabin and Brownson [18] define capacity as the observable, structural elements of organizations (e.g., size), while readiness reflects more broadly the degree to which an organization is willing and prepared to address an issue [19]. Thus, readiness includes capacity as observed and, for characteristics that cannot be easily observed, as perceived by those who complete the capacity assessment. Constructs of readiness such as resources and leadership engagement map into the “inner setting” factors of the Consolidated Framework for Implementation Research (CFIR) and are thought to influence the success of implementing a health promotion program [20]. Few studies have assessed church readiness to engage in health promotion [21, 22] and empirical data to support existing theoretical formulations such as CFIR are lacking [23].

We assessed the readiness of 100 African American churches in South Los Angeles to implement health promotion programs. In addition to obtaining local data to inform future research, we were interested in two research questions:

1. What is the readiness of small, medium and large churches? When churches participate in a randomized trial, they are often stratified by size, based on the assumptions that larger churches have more resources and may offer more activities to parishioners than smaller churches [24–27]. However, data to substantiate these assumptions are lacking.
2. Which church characteristics are associated with the implementation of wellness activities? A better understanding of these factors can inform future intervention research in church settings.

## Methods

Community and academic members of our team reviewed existing organizational readiness assessments and theoretical formulation to inform our assessment instrument [19, 20, 28–31]. Based on these articles, we developed an outline of domains, definitions of these domains and sample questions for a readiness assessment. This outline was discussed with 4 members of the clergy (two senior pastors, the chair of a health and wellness ministry and a minister). Specifically, we asked about the importance of assessing each domain, how best to phrase questions, and what would/would not be appropriate to include in the assessment. They also provided feedback on the recruitment of pastors to participate in the survey and appropriate incentives, and reviewed the draft assessment instrument before it was finalized (see Additional file 1).

In 2017, we conducted surveys with leaders of 100 churches in South Los Angeles. We started to recruit churches to which our research team had existing relationships. These churches then referred us to additional churches (snowball sampling). All surveys were conducted by one of the authors (RS) who is an African American pastor in South Los Angeles. Surveys were conducted face-to-face (32%) or by telephone (68%) with senior pastors (one per church) and lasted on average 48 min. Only one pastor refused to participate and two could not be contacted after multiple attempts. After completing the survey, several pastors commented that they appreciated the opportunity to participate in the readiness assessment and that several questions alerted them to opportunities for wellness promotion in their church. Pastors received a thank you letter with a $100 incentive after completion of the survey. Since we only collected church-related information, and not the church leaders’ private information, the need for ethics approval was deemed unnecessary by the University of California Los Angeles Institutional Review Board because the project does not involve “human subjects” as defined in the federal regulations [45 CFR 46.102(f)]. Therefore, no informed consent was obtained.

### Assessment instrument

The final questionnaire was 10 pages and assessed the following:

- Church history of wellness activities in the last 12 months; this questions was limited to the last 12 months to improve recall.
- Interest in addressing 13 specific health issues rated from 1 = not interested to 10 = extremely interested;
- Church leadership for implementation of wellness activities: Four items assessed how often the church leadership emphasized the importance of physical health at church, openly supported wellness activities, actively encouraged parishioners to participate in wellness activities and openly acknowledged the contributions of volunteers (never or rarely, 1–3 times a month, once a week, several times a week). Three items assessed to what extent the church leadership was involved in planning wellness activities, implementing wellness activities and problem solving (never or rarely, to a moderate extent, to a great extent, to a very great extent). Each item was scored from 1 to 4. Scores for all 7 items were averaged to compute the Church Leadership Score.
- Church resources to implement wellness activities, including having a budget for health- related activities, number of volunteers, having a health ministry, having a kitchen and meeting rooms, and church partnerships with clinics or outside resources that could assist in wellness efforts.
- Church willingness to implement a list of 14 activities: e.g., to ask church members to give a testimonial related to their experience with cancer or cancer screening; or to promote health as part of a research project in partnership with an academic institution (rated from 1 = not willing to 10 = extremely willing).
- Barriers to implement wellness activities: open-ended question followed by 10 probes for specific issues. Response categories were “experienced this challenge”, “expect this challenge” and “would not be a challenge”.
- Resources needed in order implement wellness activities: open-ended question, followed by 8 specific resources that were rated from 1 = not interested to 10 = extremely interested.
- Basic church characteristics including denomination; size of the active congregation, counting only members who frequently attend church events; years the church had been in operation; years that the current church leader had been at this church; number of paid staff; prior partnerships with academic institutions to promote wellness; and the existence of health and wellness policies or guidelines for healthy church meals.

### Statistical analysis

Analyses were conducted using SAS 9.4. Churches were categorized as small (less than 50 active members), medium (50–99 active members) or large (> 100 active members). These categories were recommended by two members of the research team who had been active in South Los Angeles churches for three to four decades as a pastor or first lady. Church characteristics were compared among these 3 categories (see Table 1). The Organizational Leadership Scale had a Cronbach’s alpha of 0.80, suggesting that the items have relatively high internal consistency. A Wellness Activity Score was computed as the sum of the 10 items listed in Table 2.

**Table 1:** Church characteristics by church size (number of active members < 50; 50–99; ≥ 100)

	Total (*N* = 100)	Small (*N* = 19)	Medium (*N* = 37)	Large (*N* = 44)	*P*-value
% commuters (Mean + s.d.)	29 ± 29	28 ± 35	36 ± 31	24 ± 25	NS
% long-time members	61 ± 27	64 ± 32	60 ± 28	61 ± 22	NS
years in operation (range 1–120)	54 ± 32	35 ± 29	47 ± 31	69 ± 29	< 0.0001
years of this leader at church (range 0.5–58)	16 ± 12	13 ± 8	17 ± 13	15 ± 13	NS
number of paid staff (range 0–35)	5 ± 5	2 ± 2	3 ± 2	8 ± 6	< 0.0001
# of wellness activities conducted in last 12 months (range 0–5)	1.9 ± 1.4	1.2 ± 1.1	1.8 ± 1.5	2.2 ± 1.3	< 0.05
# of barriers to implement wellness activities (experienced or expected, range 0–8)	4.8 ± 1.8	5.3 ± 1.9	4.6 ± 1.6	4.8 ± 2.0	NS
Church Leadership Scale (7 items; range 1–4)	2.6 ± 0.7	2.5 ± 0.8	2.4 ± 0.6	2.7 ± 0.6	0.09
% of churches					
in which members know each other very well	59	74	65	48	0.10
with a health/wellness ministry	41	21	30	59	< 0.01
Denomination					
Methodist	13	5	14	16	0.10
Baptist	50	42	41	61	
Non-denominational/Other	37	53	46	23	

Church size ranges from 10 to 800 active members

Chi-square test was used for categorical variables and Kruskal-Wallis tests was used for continuous variables

**Table 2:** Wellness activities conducted by church size (number of active members < 50; 50–99; ≥ 100)

Proportion of churches that …	Total (*N* = 100)	Small (*N* = 19)	Medium (*N* = 37)	Large (*N* = 44)	*P*-value
conducted > 1 wellness activity in last 12 month	56%	37%	54%	66%	0.10
ever partnered with academia to promote wellness	53%	42%	46%	64%	NS
has health advisory program	30%	16%	22%	43%	< 0.05
has health or wellness policies or goals for congregation	34%	11%	38%	41%	0.05
has guidelines for healthy church meals	37%	16%	46%	39%	0.08
promoted physical activity from the pulpit in last 12 months	87%	79%	89%	89%	NS
promoted good nutrition from the pulpit in last 12 months	90%	84%	92%	91%	NS
has partnership with clinics or outside resources that could assist in wellness efforts at church	66%	68%	59%	70%	NS
has 3 or more individuals that function as wellness champions	67%	37%	65%	82%	< 0.01
has high church leadership engagement to promote wellness	50%	42%	41%	61%	NS
Total Wellness Activity Score (range 0–10), Mean ± S.D.	5.7 ± 2.5	4.3 ± 2.4	5.5 ± 2.3	6.5 ± 2.5	< 0.01

Total Wellness Activity Score was computed as the sum of the 10 items listed in the table

Chi-square test was used for categorical variables and Kruskal-Wallis tests was used for continuous variables

To assess potential bias due to the mode of survey administration, we compared responses between face-to-face versus telephone surveys on four key variables, including Church Leadership Score, Wellness Activity Score, number of wellness activities conducted in the last 12 months and number of barriers to implement wellness activities. We found statistically significant differences in number of wellness activities conducted in the last 12 months (2.3 + 1.5 reported in face-to-face surveys versus 1.7 + 1,3 reported in phone surveys, *p* < .05). Therefore, we combined responses from face-to-face and telephone surveys and controlled for mode of administration when needed. Based on examination of the same four key variables (data not shown), we grouped denominations into 3 categories: Methodist (including United Methodist and African Methodist Episcopal churches), Baptist and non-denominational/other denominations.

Finally, we mapped survey items onto some of the inner setting domains of the CFIR theoretical framework [20] and explored their relationship to the number of wellness activities reported by church leaders. Similar to prior research [32], we conceptualized church policies regarding healthy church meals and prior partnership with academia to promote wellness at church as indicators of church social norms and values which align with the CFIR definition of “*Culture*”. We also considered denomination since there may be variations with respect to social norms, values and traditions regarding healthy lifestyles that may affect health promotion at church. We also mapped survey items into the constructs of “*Structural Characteristics*”, “*Leadership Engagement”*, “*Resources*” and “*Networks and Communication*”. We conducted a series of regression analyses to determine to what extent particular church-related variables and constructs of the CFIR inner setting explained the amount of variation of number of wellness activities conducted in the last 12 months. The analyses for the full linear regression model controlled for method of survey administration. Base-10 logarithm or power transformations were used on skewed variables before regression analysis was applied. Influence analyses that involved refitting the model without potentially influential observations showed that the inferences were robust. Diagnostics indicated that variables were modestly correlated and that there was minimal collinearity (highest variance inflation factors were < 2.5).

## Results

Members of the clergy provided the following suggestions: calling the assessment a church *readiness* assessment instead of a *capacity* assessment, which was perceived as being more judgmental; introducing the assessment by explaining health disparities in South LA to justify the need for this research; having a mix of open-ended and closed-ended questions to give church leaders the opportunity to explain the special circumstances at their respective churches in their own words; asking for churches’ *interests* to implement specific wellness activities rather than their *willingness*; asking questions about churches *implementing* wellness activities versus *hosting* a wellness program that would be delivered by an outside expert; and asking about churches’ interest in receiving specific types of support for implementing wellness activities that researchers could realistically provide, such as providing print information from reputable sources for distribution to church members, or a list of local resources that would help churches to refer parishioners who have questions or need services.

The church leaders also emphasized the importance of a “warm introduction” to obtain a good participation rate among church leaders, offered to send a text or e-mail to introduce our team to their colleagues, and recommended a $100 incentive for ministers who completed the survey.

### Church characteristics

Fifty percent of the churches in the sample were Baptist, 13% were Methodist, and 37% were non-denominational or of another denomination (see Table 1). About 30% of churchgoers were commuters who only attended religious services on the weekend but no other church activities, and about 60% were long-time members. On average, the churches had been in operation for 54 years and leaders completing the assessment had been affiliated for 16 years. Churches had on average 5 paid staff, with a wide range from 0 to 35. About 40% of the churches had a health or wellness ministry. Small, medium and large churches had similar proportions of long-time members and commuters.

On average, large churches had the highest number of paid staff, were most likely to have a health or wellness ministry and had been in operation for the longest time. Medium and large churches conducted significantly more wellness activities than small churches (all *p* < 0.05). In small churches, members tended to be more likely to know each other very well compared to large churches. Baptist churches tended to be large and non-denominational/other churches tended to be small (both *p* = 0.10).

### Wellness activities conducted by churches

Eighteen percent of churches did not conduct any wellness activities in the last 12 months, 26% conducted one activity, 25% conducted two activities, 18% conducted three activities and 13% conducted four or five activities. Wellness activities in the last 12 months addressed cancer (39%), coronary vascular disease/stroke (29%), physical activity (22%), diabetes (17%) and nutrition (21%). In addition to these issues, many churches were interested in addressing violence in the community (73%), domestic violence (60%) and preventing sexually transmitted infections (60%). More than half of the churches had ever partnered with an academic institution to promote wellness at their church (see Table 2). Only about one third of the churches had a health advisory program, guidelines for healthy church meals, or health or wellness policies or goals for the congregation. The majority of church leaders reported that they promoted physical activity and good nutrition from the pulpit and 2/3 of them reported partnerships with clinics or outside resources, with no differences by church size. Based on a modified Implementation Leadership Scale, church leaders were moderately involved in supporting wellness activities.

Large churches were significantly more likely than small and medium churches to have a health advisory program, health or wellness policies or goals for the congregation and 3 or more individuals who function as wellness champions (all *p* < .05). The Total Wellness Activity Score that was computed from all variables reported in Table 2 was significantly different by church size, with larger churches scoring higher than smaller churches.

### Barriers to implementing wellness activities

Since only 31% of the churches had a budget for health-related activities and of those, 52% stated that it was insufficient, 85% of churches had an insufficient budget. Regardless of church size, insufficient budget was the most commonly cited barrier to implementing wellness activities (85%), followed by lack of other resources (81%). A substantial proportion of churches was not sure how to implement wellness activities (61%) and lacked volunteers (58%). Almost half of the churches had either experienced or expected that their members were not interested in wellness activities (47%). Thirty-seven percent were not sure what topics would be of interest to their members. More than one third of churches lacked a commitment from the leadership. Some churches (32%) had too many activities already ongoing, especially among large churches (43%). Only 24% of all churches had concerns that members did not like to participate in research, but a higher proportion of small churches (37%) tended to have this concern. Small churches were significantly more likely to report size of membership as a barrier to implementing wellness activities compared to medium and large churches (*p* < 0.01). Other than that, there were no statistically significant differences in barriers by church size and the number of barriers was similar for small, medium and large churches (see Table 3).

**Table 3:** Churches that experienced or expect barriers to implementing wellness activities by church size (number of active members < 50; 50–99; ≥ 100)

	Total (*N* = 100)	Small (*N* = 19)	Medium (*N* = 37)	Large (*N* = 44)	*p*-value
Insufficient budget	85%	95%	84%	82%	NS
Lack of other resources	81%	84%	78%	82%	NS
Not sure how to implement wellness activities	61%	63%	62%	59%	NS
Not enough volunteers	58%	53%	62%	57%	NS
Members not interested	47%	63%	38%	48%	NS
Not sure what topics members would be interested in	37%	32%	38%	39%	NS
Lack of commitment from church leadership	36%	37%	30%	41%	NS
Too many activities already ongoing	32%	16%	27%	43%	0.07
Members don’t like to participate in research	24%	37%	22%	20%	NS
Size of membership	21%	47%	19%	11%	< 0.01
# of barriers to implement wellness activities (Mean ± standard deviation)	4.8 ± 1.8	5.3 ± 1.9	4.6 ± 1.6	4.8 ± 2.0	NS

Chi-square test was used for categorical variables and Kruskal-Wallis tests was used for continuous variables

### Resources to implement wellness activities

Most churches had meeting rooms, a kitchen and a few dedicated volunteers. Overall, churches were most interested in obtaining the following resources: Gift cards for volunteers or study participants; a list of local resources to which to refer members who have questions or need help; printed health information; a list of speakers for church events; a sample of a needs assessment; and workshops to inform volunteers so they can inform members. All of these resources were rated between 9 and 10 on a scale from not interested [1] to extremely interested [10] with no differences by church size.

### Churches’ willingness to conduct selected activities

As shown in Table 4, Churches were highly willing to implement low-intensity strategies such as distributing print information, but they were also quite willing, on average, to conduct surveys with parishioners, identify volunteers, and to institute church policies to promote health. They also expressed strong interest in conducting health fairs at churches. Willingness to implement these activities did not differ by church size.

**Table 4:** Church willingness to conduct selected activities (*N* = 100)

Activity (10 point scale from not willing (1) to extremely willing (10)	M ± S.D.
Distribute print information on various health topics	9.5 ± 1.3
Plan and conduct a health fair at your church	9.3 ± 1.3
Partner with an academic institution to promote health	9.3 ± 1.5
Host a speaker and advertise the event at your church	9.2 ± 1.5
Survey or debrief parishioners to determine the success of a wellness activity	9.0 ± 1.7
Conduct a survey with parishioners to identify health concerns	8.9 ± 1.6
Host a health program at your church and help to recruit members, but the program itself would be delivered by an outside expert	8.9 ± 1.6
Promote health as part of a research study	8.8 ± 1.8
Identify volunteers who would be trained to provide counseling	8.5 ± 2.1
Ask church members to give a testimonial	8.3 ± 2.0
Raise funds to support a wellness activity at your church	8.2 ± 2.2
Regularly incorporate health messages into the sermon	8.1 ± 2.1
Institute policies regarding the food that can be served at church	7.7 ± 2.4
Incorporate 5–10 min exercise breaks into church activities	7.1 ± 2.6

Mean ± Standard deviation; only shown for all churches combined since there were no significant differences by church size

### Exploring inner setting factors associated with implementation of wellness activities

In bivariate analyses, 12 items of the inner setting constructs were significantly associated with number of wellness activities conducted in the past 12 months in the expected direction (see Table 5). For example, in the domain of structural characteristics, larger churches that had been in operation for more years and had a larger number of paid staff had implemented more wellness activities in the past 12 months. With respect to organization norms and values, Methodist churches and churches that had ever partnered with academia to promote wellness and had guidelines for healthy church meals had also implemented more wellness activities in the past 12 months. Churches with higher leadership engagement scores and those that had partnerships with clinics or outside resources reported more wellness activities, as well as churches with more resources (health/wellness ministry, number of volunteers, sufficient budget) and fewer barriers. Churches that completed the readiness assessment face to face reported significantly more wellness activities than those who completed the assessment by phone. The two items that were not associated with this outcome were: number of years the current leader had been at the church and “church has health or wellness policies or goals for the congregations.” (Data not shown). Bivariately, church characteristics that explained most of the variation in this outcome were number of paid staff (R^2^ = 16%), denomination (R^2^ = 14%), has a health/wellness ministry (R^2^ = 13%), size of active congregation (R^2^ = 11%), number of volunteers (R^2^ = 11%) and having a budget for health-related activities (R^2^ = 11%).

**Table 5:** Relationships between church characteristics sorted by the Inner Settings of the Consolidated Framework for Implementation Research (Damschroder et al., 2009) and number of wellness activities conducted by African American churches in the past 12 months

Church Characteristics	Bivariate relationship between each item and outcome			Adjusted relationship between domain and outcome			Full Model R-square = 0.45	
Items	β	P	R^2^	β	P	R^2^	β	P
**Structural Characteristics** (Organization social architecture, age, maturity and size)								
Size of active congregation^a^	1.11	< 0.01	0.11	0.36	NS	0.18	0.19	NS
Years church has been in operation	0.01	< 0.01	0.08	0.005	NS		0.008	0.09
Number of paid staff^a^	1.60	< 0.01	0.16	1.13	0.02		1.05	< 0.05
**Culture** (Organization norms and values								
Denomination (reference group: non-denominational/other)								
Methodist	1.10	< 0.01	0.14	0.99	0.02		0.32	NS
Baptist	−0.50	0.08		−0.58	0.03	0.25	−0.94	< 0.01
Ever partnered with academia to promote wellness at church 1 = yes, 0 = no	0.64	0.02	0.05	0.46	0.07		0.01	NS
Has guidelines for healthy church meals 1 = yes 0 = no	0.76	< 0.01	0.07	0.77	< 0.01		−0.25	NS
**Leadership Engagement** (Commitment, involvement and accountability of leaders with the implementation)								
Church leadership scale, 7 items	0.59	< 0.01	0.08	–			0.05	NS
**Resources** (level of resources dedicated for implementation and ongoing operations, including money, training, education, physical space, time)								
Has a health/wellness ministry? 1 = yes,0 = no	1.01	< 0.01	0.13	0.46	NS	0.23	−0.01	NS
Number of members who frequently volunteer^a^	1.20	< 0.01	0.11	0.60	0.10		−0.17	NS
Has a budget for health-related activities 1 = yes, 0 = no	1.01	< 0.01	0.11	0.50	NS		−0.47	NS
# Barriers to implement wellness activities, experienced or expected^b^	−0.13	< 0.01	0.09	−0.09	0.03		−0.07	NS
**Networks and Communication** (The nature and quality of webs of social networks)								
Has partnerships with clinics or outside resources that could assist in wellness efforts 1 = yes, 0 = no	0.83	< 0.01	0.08	–			−0.36	NS
**Method of survey administration** 1 = face-to-face, 0 = phone	.60	0.04	0.04	–			−0.18	NS

^a^Log 10 transformation was used

^b^1.25 power transformation was used

Domains are in boldface

Church culture (3 items) and resources (4 items) were the most important inner setting domains, each explaining 23–25% of variation, followed by structural characteristics, which explained 18% of the variation. It should be noted that two of the domains were assessed with single items or scores (leadership engagement and networks and communication), which may have contributed to a low R square for these two items (8% each). The full model that included all 12 items and controlled for mode of survey administration explained 45% of the variation in number of wellness activities conducted in the past 12 months, providing empirical evidence that the inner setting is indeed a very important component of the CFIR. In the full model, in which all items were considered, number of paid staff and denomination were significantly associated with the outcome. The coefficients for these 2 variables indicate that, on average and after controlling for all other variables in the model, the number of wellness activities conducted in the last 12 months increased by one for each additional paid staff at a church and it was decreased by about one in Baptist churches as compared to non-denominational/other denomination churches.

## Discussion

This study provides local data on the readiness/capacity of African American churches in South Los Angeles to engage in health promotion activities. With a focus on understanding church level factors, our findings complement studies that examine the role of pastors of African American churches [2, 33], the characteristics of church members who are willing to attend health promotion activities [34] and the association between health promotion activities at churches and health behaviors and intentions of parishioners [35]. Our study also builds on and expands previous research with African American churches that focused on instrument development to assess church capacity [21, 30]. Similar to other studies [21, 30, 36], our findings highlight the importance of active support and public endorsement of health-related activities by the pastor and of having appropriate resources to implement health-related activities. The importance of resources cannot be stressed enough, especially if churches are located in disadvantaged and under-resourced communities such as South Los Angeles, which has been identified as a high poverty area [37].

### Church characteristics associated with implementation of wellness activities

Overall, we found statistically significant differences between small, medium and large churches with respect to number of wellness activities conducted and with respect to resources (e.g., having a health/wellness ministry; number of paid staff). These findings confirm the need for stratification of churches by size when conducting intervention research. A total of 12 church characteristics were bivariately associated with number of wellness activities conducted by churches in the last 12 month. Although we did not assess *all* inner setting constructs (e.g. implementation climate), the 12 items combined explained a substantial amount of variation in number of wellness activities implemented in the last 12 month. Interventions in church settings often achieve moderate outcomes, especially if intervention components are only partially implemented or if the study addresses a lifestyle behavior that is difficult to modify [38, 39]. In an experimental or quasi-experimental design, imbalances between study groups in any of the inner setting factors of the CFIR could mask or magnify a relatively small change in study outcome. Since it is not possible to stratify on all of these factors, we recommend a comprehensive assessment of church characteristics at study onset, so any imbalances can be considered during group assignment and/or data analysis.

### Assessment of “culture” in church settings

Organizational culture has been defined as “norms, values, and basic assumptions of a given organization” [20] and is thought to be relatively stable [40]. It has been assessed in organizations such as worksites or clinics using items such as “People at all levels openly talk about what is and isn’t working” or “People in this clinic operate as a real team” [41]. Responses from pastors to these and similar items may be highly susceptible to reporting bias and social desirability bias. In one church study, climate was operationalized as “church leaders providing space for a program, making announcements about the program and demonstrating knowledge of the program towards parishioners” [42], which overlaps with the construct of leadership engagement and support. Existing measures of organizational culture are lengthy and lack validation [43], especially in church settings. In light of these limitations, we considered denomination, prior partnerships with academic institutions to promote wellness, and the existence of health and wellness policies or guidelines for healthy church meals as a reflection of church culture.

In bivariate analyses and in the final model that included items of five inner settings constructs, denomination was significantly associated with number of wellness activities conducted in the past 12 months. This is a new finding and has to be interpreted with caution until is it replicated in other studies. Historically, African American churches have been engaged in health promotion activities to address health disparities in their communities and both the National Baptist Convention and the African Methodist Episcopal Church have developed national health ministries to serve the health and wellness needs of their congregations [2]. We recommend to further examine the role of denomination in future intervention research in faith-based settings.

### Limitations

Limitations of this study include a cross-sectional design. Although several church characteristics are relatively stable and can be considered as predictors of wellness activities reported by pastors (such as size of congregation, number of paid staff), other characteristics could be a *result* of conducting wellness activities such as developing a partnership with clinics or outside resources. Church characteristics were reported by one person per church, mostly senior pastors, who may have wanted to present the best possible image of their church and of their support for promoting wellness activities. Therefore, responses may suffer from social desirability bias. We attempted to limit biases by carefully vetting questions with pastors during the development of the assessment instrument, and by asking concrete questions regarding the frequency of pastors’ activities rather than asking for a self-assessment of how supportive they are. Respondents appeared to be outspoken in reporting challenges to implement wellness activities, including lack of commitment from church leadership. In addition, we had to combine many different denominations into the “other” category due to small numbers and this category was combined with non-denominational in the analysis. We analyzed a convenience sample that was limited to churches in South Los Angeles; however, our sample reflects that the majority of African Americans consider themselves Baptist (45%) or Methodist (12%) [44].

## Conclusions

Many African American churches in South Los Angeles are already actively engaged in health promotion activities or interested in conducting health promotion in the future, despite a general lack of resources. We collected local data that identified the needs of churches regarding resources and their preferences regarding health promotion activities that will guide our future collaborative health research in South Los Angeles. In our sample, only about one third of the churches had health or wellness policies. Since policy changes can potentially reach all church members [45] and since leaders were quite willing to implement them, we recommend promoting implementation of health policies in future health interventions. Our findings suggest the need for a comprehensive assessment of church characteristics in intervention studies to avoid imbalances that can mask or magnify study outcomes. In addition, our data support the importance of CFIR inner setting constructs for the implementation of wellness activities in African American churches.

## Additional file

Additional file 1: Church Readiness Assessment Questionnaire. (PDF 513 kb)

## Abbreviations

CFIR: Consolidated Framework for Implementation Research
CHA: Community Health Advisor
