# Supplementary material for: Organizational readiness for wellness promotion – a survey of 100 African American church leaders in South Los Angeles
Source: BMC Public Health. 2019 May 17;19:593. doi: 10.1186/s12889-019-6895-x (PMC6525409; doi:10.1186/s12889-019-6895-x)
Supplement: Supplementary file 1 — Church Readiness Assessment Questionnaire. (PDF 513 kb) [file 12889_2019_6895_MOESM1_ESM.pdf]

Today's Date: ____________ Time start/end: _________/_________ Phone Face to Face ID No. _____

Name of Church ____ Denomination ___________________

Address: ____

Name of Key Informant ___________________________________

University of California Los Angeles/Charles Drew University Partnership to Eliminate Cancer Health Disparities

NIH/NCI# U54 CA 143931

Outreach Core Leaders: Annette E. Maxwell, Dr.P.H., Aziza Lucas-Wright, M.Ed.

Readiness for Wellness Promotion among Churches in South LA

(READ CONVERSATIONAL, USE PASTOR'S NAME)

Hello, may I speak to ___________________. My name is _____________. Pastor XYZ referred me to you. I am a member of the Charles Drew University/UCLA research team. Our team is partnering with churches in South Los Angeles to promote health in the community. We know that many churches are committed to improving the spiritual, emotional <u>and</u> physical health of their parishioners and our team would like to talk to the leadership of 100 churches in South LA to find out how we can support this effort. I would like to talk to you for 40-60 minute and I am able to give you a token of $100 to show our appreciation.

Pastor x, we are focusing on cancer because there are many cancer disparities in South LA. For example, African American women are more likely to die from breast cancer, cervical cancer and colorectal cancer than any other group in the United States. African American men are more likely to die from prostate cancer than any other group. We would like to ask questions about the health needs in your community and your church's interests, readiness and resources to address these health needs. Having data on the churches' perceived needs and interests, and what type of support churches would find helpful if they partner with us in cancer control efforts will guide our future efforts to reduce health disparities. Is it ok to talk now?

YES NO → THANK HIM/HER FOR THEIR TIME AND ASK IF THEY WOULD LIKE TO SHARE WITH YOU WHY THEY DECLINE TO ANSWER THE QUESTIONS __________________________
Are you ready for us to begin the interview now? (CIRCLE ONE NUMBER)

1 YES → Thank you for participating in this study. Your participation is voluntary. You may stop the interview at any time and refuse to answer any question. GO TO PAGE 2

2 NO

Would you like to re-schedule this follow-up interview? (CIRCLE ONE NUMBER)

1 YES → What date and time would be convenient for us to call again for the interview?

DATE AND TIME______________________________

2 NO. → Thank you for your time. Good bye.

A. CHURCH HISTORY OF WELLNESS ACTIVITIES

First, I would like to find out if your church has been involved in any wellness activities in the last 12 months and what type of activities these were. These could be activities to promote mental health, better nutrition, physical activity, flu shots, cancer screening, cancer support groups, blood pressure screening, HIV/AIDS, or any other health issues.

(OPEN ENDED QUESTION. TAKE NOTES ON HEALTH ISSUE ADDRESSED AND FORMAT: WHAT WAS DONE? AFTER EACH ACTIVITY ASK: ANY OTHER ACTIVITIES?)

**Health Issue addressed**	**Format: What was done?**

IF MORE THAN 3 WELLNESS ACTIVITIES, NOTE ON THE BACK OF THE PAGE

B1. Have you ever partnered with an academic institution to promote wellness at your church?

1. YES
2. NO [GO TO C]

IF YES: B2.With which institution? ____

C. CHURCH INTERESTS IN IMPLEMENTING WELLNESS ACTIVITIES (HEALTH ISSUES)

We realize that there are a number of health issues that your church could address. I will read a list of health issues and would like you to rate how interested your church would be in implementing activities to address these health issues on a scale of 1 to 10 with *1 being not interested* and *10 being extremely interested.* Please answer these questions regardless of programs that you have implemented in the past or that you are currently implementing. E.g., if your church is extremely interested in decreasing alcohol intake, you should rate it as "10" even if your church is already addressing this health problem.

Not interested — Extremely interested

**How interested would your church be to implement activities for….**	**1**	2	3	4	5	6	7	8	9	**10**
C1. Decreasing alcohol intake?	**1**	2	3	4	5	6	7	8	9	**10**
C2. Stopping smoking?	**1**	2	3	4	5	6	7	8	9	**10**
C3. Encouraging physical activity?	**1**	2	3	4	5	6	7	8	9	**10**
C4. Encouraging healthy eating?	**1**	2	3	4	5	6	7	8	9	**10**
C5. Obtaining flu shots?	**1**	2	3	4	5	6	7	8	9	**10**
C6. Having boys and girls vaccinated for Human Papilloma Virus (HPV) to prevent several types of cancer?	**1**	2	3	4	5	6	7	8	9	**10**
C7. Preventing sexually transmitted diseases?	**1**	2	3	4	5	6	7	8	9	**10**
C8. Screening for diabetes?	**1**	2	3	4	5	6	7	8	9	**10**
C9. Screening for hypertension?	**1**	2	3	4	5	6	7	8	9	**10**
C10. Promoting cancer screening?	**1**	2	3	4	5	6	7	8	9	**10**
C11. Preventing or living with HIV/AIDS	**1**	2	3	4	5	6	7	8	9	**10**
C12. Dealing with violence in the										

family	**1**	2	3	4	5	6	7	8	9	**10**
C13. Dealing with violence in the community	**1**	2	3	4	5	6	7	8	9	**10**

C14. Can you think of any other health issues that your church would like to address? (SPECIFY)

____

D. CHURCH LEADERSHIP FOR IMPLEMENTATION OF WELLNESS ACTIVITIES

The following questions are about how church leaders (IF PASTOR IS THE RESPONDENT, SAY: "you") may promote wellness activities at your church.

D1. How often does the church leadership emphasize the importance of physical health among parishioners? Would you say ….

1. Never or rarely
2. 1-3 times a month
3. Once a week or
4. Several times a week
8. DK
9. RF

D2. How often does the church leadership *openly support wellness activities* at your church when talking to parishioners or volunteers? Would you say ….

1. Never or rarely
2. 1-3 times a month
3. Once a week or
4. Several times a week
8. DK
9. RF

D3. How often does the church leadership *actively encourage parishioners or volunteers to participate in wellness activities* at your church? Would you say….

1. Never or rarely
2. 1-3 times a month
3. Once a week or
4. Several times a week
8. DK
9. RF

D4. How often does the church leadership *openly acknowledge the contributions of volunteers*? Would you say...

1. Never or rarely
2. 1-3 times a month
3. Once a week or
4. Several times a week
8. DK

9. RF

D5. To what extent is the church leadership involved in *planning* wellness activities at your church? Would you say……

1. Never or rarely
2. To a moderate extent
3. To a great extent or
4. To a very great extent
8. DK
9. RF

D6. To what extent is the church leadership involved in *implementing* wellness activities at your church? Would you say ….

1. Never or rarely
2. To a moderate extent
3. To a great extent or
4. To a very great extent
8. DK
9. RF

D7. To what extent is the church leadership involved in *problem solving* for the implementation of wellness activities? Would you say….

1. Never or rarely
2. To a moderate extent
3. To a great extent or
4. To a very great extent
8. DK
9. RF

CHURCH RESOURCES TO IMPLEMENT WELLNESS ACTIVITIES

The next questions are about the resources of your church to implement wellness activities.

D8. Does your church have a person appointed who is responsible for health-related activities?

1. YES 2. NO 8. DK 9. RF

D9. Health advisors are trained individuals who can give advice or information on specific health issues. Does your church have a health advisor program?

1. YES 2. NO 8. DK 9. RF

D10. Does your church have a budget for health related activities?

1. YES 2. NO 8. DK 9. RF

IF YES:

D11. Would you say your church budget is

1. not sufficient
2. enough to support *some* of the expenses for wellness activities or

3. sufficient to cover *all or most* of the expenses for wellness activities
8. DK 9.RF

D12. Does your church have other resources for health-related activities, such as volunteers, a kitchen, meeting rooms, etc.? Please list the resources: ____

____

____

D13. Volunteers who have a passion to promote wellness are also called "Wellness Champions" and can be a tremendous asset for promoting wellness in churches. Does your church have individuals that you would describe as wellness champions?

1. YES → D14. How many? __________
2. NO
8. DK 9. RF

D15. Has your church established health or wellness policies or goals for the congregation?

1. YES 2. NO 8. DK 9. RF

IF YES:
D15A. What are these policies or goals? ____

D16. In the past 12 months have you or another minister (has your pastor or other minister) promoted the importance of physical activity in a sermon or Pastor's Words?

1. YES 2. NO 8. DK 9. RF

D17. In the past 12 months, have you or another minister (has our pastor or other minister) promoted the importance of good nutrition in a sermon or Pastor's Words?

1. YES 2. NO 8. DK 9. RF

D18. Does your church have guidelines for healthy church meals?

1. YES 2. NO 8. DK 9. RF

D19. Does your church have partnerships with clinics or outside resources that could assist in wellness efforts at your church?

1. YES 2. NO 8. DK 9. RF

IF YES:
D20. Who are your partners? ____

D21. How likely do you think your parishioners will participate in wellness activities at your church? Would you say….

1. Not likely
2. Somewhat likely
3. Very likely

8. DK
9. RF

DELIVERY FORMAT FOR WELLNESS ACTIVITIES OR PROGRAMS

Churches can engage in health promotion in different ways, from encouraging a healthier lifestyle *from the pulpit*, to distributing *print information*, participating in *health fairs* or doing *workshops* for parishioners on specific issues. I will read a list of wellness activities and would like you to rate how willing your church would be to implement these activities on a scale of 1 to 10 with 1 being not willing and 10 being extremely willing to implement the activity. Please answer these questions considering the resources you have at your church and assuming that you would receive some support to get started.

	Not willing									Extremely willing
**How willing would your church be to do the following:**										
D22. Regularly incorporate health messages into the sermon or Pastor's Words?	**1**	2	3	4	5	6	7	8	9	**10**
D23. Ask church members to give a testimonial?	**1**	2	3	4	5	6	7	8	9	**10**
D24. Institute church policies regarding the food that can be served at church events?	**1**	2	3	4	5	6	7	8	9	**10**
D25. Incorporate 5-10 minute exercise breaks into church activities (e.g., choir practice)	**1**	2	3	4	5	6	7	8	9	**10**
D26. Conduct a survey with parishioners to identify health concerns at your church?	**1**	2	3	4	5	6	7	8	9	**10**
D27. Identify volunteers who would be trained to provide counseling for parishioners?	**1**	2	3	4	5	6	7	8	9	**10**
D28. Host a health program at your church and help to recruit parishioners, but the program itself would be delivered by an outside expert.	**1**	2	3	4	5	6	7	8	9	**10**
D29. *Host* a speaker and advertise the event at your church?	**1**	2	3	4	5	6	7	8	9	**10**
D30. Distribute print information on various health topics?	**1**	2	3	4	5	6	7	8	9	**10**
D31. Plan and conduct a health fair at your church?	**1**	2	3	4	5	6	7	8	9	**10**
D32. Partner with an academic institution to promote health?	**1**	2	3	4	5	6	7	8	9	**10**
D33. Promote health as part of a *research* project in partnership with an academic institution?	**1**	2	3	4	5	6	7	8	9	**10**

D34. Conduct a survey or debriefing of parishioners to determine the success of a wellness activity	**1**	2	3	4	5	6	7	8	9	**10**
D35. Raise funds to support a wellness activity at your church	**1**	2	3	4	5	6	7	8	9	**10**

D36. Is your church (CIRCLE ONE)

1. interested in planning and creating your own wellness activities or
2. more interested in implementing wellness activities that have been used successfully in other churches?

8. DK 9. RF

E. BARRIERS TO IMPLEMENT WELLNESS ACTIVITIES

We realize that it takes resources, time, volunteers and dedication to implement wellness activities at your church, to promote them among parishioners and to keep them going. What are some of the challenges that you have experienced in your church or that you would expect in your church?

(OPEN ENDED, WRITE DOWN RESPONSES, NOTE IF CHALLENGES HAVE BEEN EXPERIENCED OR ARE EXPECTED, PROBE FOR EVERYTHIN IN THE TABLE)

Type of challenge (PROBE: WHAT ABOUT …)	Experienced Challenge	Expect Challenge	Would not be a challenge	Notes
Size of membership<br>Too big?<br>Too small?				
Members not interested				
Not enough volunteers				
Lack of other resources				
Insufficient budget				
Too many activities going on already				
Not sure how to implement wellness activities				
Not sure what topics members would be interested in				

Lack of commitment from church leadership				
Our members don't like to participate in any type of research.				

IF MORE BARRIERS, NOTE ON THE BACK OF THE PAGE

F. SUPPORT AND RESOURCES TO IMPLEMENT WELLNESS ACTIVIITES

The following questions are about additional resources and support that your church would need to implement wellness activities. Thinking about the resources that are already available at your church, what additional resources and support would your church require to implement wellness activities? (OPEN ENDED, WRITE DOWN RESPONSES)

When researchers partner with churches, they are often able to provide some resources to the church. Knowing what type of resources churches would like helps us to plan what we should offer. I will read a list of resources and would like you to rate how interested your church would be in obtaining each resource on a scale of 1 to 10 with 1 being not interested (1= we don't need this) and 10 being extremely interested in obtaining this resource. A rating of 5 means "may be nice to have or moderate interest in getting this resource."

How interested would your church be in obtaining …. Not interested — Extremely interested

F1. Printed information on selected health topics from reputable sources for distribution to church members	**1**	2	3	4	5	6	7	8	9	**10**
F2. Short videos on specific health topics with discussion questions that your church could show and discuss with church members	**1**	2	3	4	5	6	7	8	9	**10**
F3. Workshops for church volunteers to provide them with information on selected health topics that they can pass on to parishioners	**1**	2	3	4	5	6	7	8	9	**10**

F4. A list of local resources for different health issues that would help your church to refer parishioners who have questions or need services	**1**	2	3	4	5	6	7	8	9	**10**
F5. Workshops for church volunteers to provide them with information on available wellness programs, how to adapt them for your church and how to implement them.	**1**	2	3	4	5	6	7	8	9	**10**
F6. A sample of a needs assessment questionnaire that your church could administer to parishioners.	**1**	2	3	4	5	6	7	8	9	**10**
F7. A list of speakers that your church could invite to give a talk and their areas of expertise	**1**	2	3	4	5	6	7	8	9	**10**
F8. Gift cards that your church could distribute to recognize individuals who assist or participate in wellness activities	**1**	2	3	4	5	6	7	8	9	**10**

G. CHURCH CARACTERISTICS

Finally, I would like to get some general information on your church.

G1. What is the size of your congregation, counting only members who frequently attend church events? _____

G2. How would you describe the make-up of your congregation?
for example when considering the entire congregation about what percent of the congregation is elderly, ______________What percent of the congregation is young families, __________________
What percent of the congregation are commuters, who usually come for worship but not for other activities? __________
What percent are long-time members versus recent members? ____________% long-time members

G3. How well do the members know each other? Would you say
1. Not well at all
2. Somewhat well
3. Very well

G4. What is the number of members who frequently volunteer? ______________

G5. How many years has this church been in operation at this or a nearby location? __________

G6. How many years has the current church leader (you) been at this church? _____________

G7. What is the number of paid staff at this church? ________________

G8. Do you have a health/wellness ministry? 1. YES 2. NO

IF YES: G9. How active would you say is your health/wellness ministry?
1. Rarely active
2. Somewhat active
3. Very active

INFORMATION ON RESPONDENT (INTERVIEWER FILL IN IF RESPONDENT IS CHURCH LEADER)

G10. About how many years have you been at this church? _____________

G11. What is your current position in your church? Circle all numbers that apply:
1. Senior Pastor
2. Minister
3. Board of Directors
4. President or Head of the Health Ministry
5. Officer of the Health Ministry
6. Committee Chair
7. Committee Member
8. Member of the church
9. Other: Specify:____

Thank you for providing this information, which will inform our future work in South Los Angeles!
